# Bovine endometrium-derived cultured cells are suitable for lipofection

**DOI:** 10.1038/s41598-021-95848-0

**Published:** 2021-08-10

**Authors:** Mai Shiokawa, Ryotaro Miura, Aki Okubo, Yujiro Hagita, Itaru Yoshimura, Hiroshi Aoki

**Affiliations:** 1 School of Veterinary Nursing and Technology, Faculty of Veterinary Science, Nippon Veterinary and Life Science University, Tokyo, 180-8602 Japan; 2 School of Veterinary, Faculty of Veterinary Science, Nippon Veterinary and Life Science University, Tokyo, 180-8602 Japan; 3 Fuji Animal Farm, Nippon Veterinary and Life Science University, Yamanashi, 401-0338 Japan

**Keywords:** Transfection, Cell culture, Virology

## Abstract

Bovine-derived cultured cells, including Madin-Darby bovine kidney cells, are used worldwide; however, lipofection tend to result in low transfection efficiency, which has impeded the progress of veterinary research. We performed experiments to confirm the lipofection efficiency of bovine-derived cultured cells, to identify cells that suitable for lipofection. Several bovine tissues (endometrium, testis, ear tissue and foetal muscle) were collected, and primary cultured cells were prepared. Lipofection assay showed that only bovine endometrium (BE)-derived cells could be transfected efficiently (50‒70%). BE cells can be divided into at least two types of cell populations (BE-1 and BE-2). The BE-1 cells, which were suitable for lipofection, were obtained by passages at short intervals and were negative for cytokeratin- and positive for vimentin-expression; the BE-2 cells did not have these characteristics and were not suitable for lipofection. Furthermore, the BE-1 cells and artificially immortalised cells of BE-1, iBE-1 cells, were utilised in a reporter assay requiring the introduction of multiple DNAs. Endometrial tissues can be collected from living cows, and BE-1 cells can be obtained easily by controlling passaging timing. The production of BE-1 cells and sharing the methods required to prepare them will contribute to the development of veterinary research.

## Introduction

The introduction of exogenous genes into cultured cells and living animals is an indispensable technique used regularly to analyse the functions of genes and gene products. Gene introduction methods can be divided broadly into three types: chemical methods (transfection), physical methods (electroporation and microinjection) and biological methods (viral vectors), and an appropriate method must be selected based on the purposes of the experiment^1^. Transfection, which is classified as a chemical method, is a very simple technique that can be performed without special equipment and is used widely to introduce genes into cultured cells. The diethylaminoethyl-dextran method^2,3^ and the calcium phosphate method^4,5^ were developed between the 1960s and the 1980s; however, recently, lipofection methods, using cationic liposomes and polymers, are being used widely^6^. Although lipofection allows the introduction of genes into various cultured cells, there are still cultured cells in which gene transfer by lipofection is difficult. When using the cultured cells that are unsuitable for lipofection, physical or biological methods must be selected; however, these methods often require specialised equipment and require additional time, such as the time needed to produce a viral vector.

The lipofection efficiency of cultured cells is often described in the product information provided by the manufacturers of lipofection reagents, allowing the transfection efficiency of targeted cells to be known prior to performing a lipofection. However, this information is often limited to cultured cells that are used frequently, especially human- and rodent-derived cultured cells. In contrast, few reports have examined the lipofection efficiency of bovine-derived cultured cells^7,8^. Osorio et al.^8^ investigated the transfection efficiency of Madin-Darby bovine kidney (MDBK) and bovine mammary epithelial alveolar (MACT) cells using lipofection^9,10^. Examinations of lipofection efficiency under various conditions have shown that the maximum efficiencies achieved by MACT and MDBK cells were 29.5 ± 1.9% and 4 ± 0.4%, respectively^8^. However, MACT cells have also been shown to have high cell mortality^8^, which may limit their use. Transfection assays were performed using cultured cells derived from bovine fibroblasts; however, no consensus has been reached regarding the lipofection efficiency in these cells^11–13^. This low transfection efficiency in bovine-derived cultured cells makes experimentation difficult, especially those that require the introduction of multiple exogenous DNA sequences, such as reporter assays and genome editing strategies, hindering the progress of veterinary research. In this study, we have succeeded in producing bovine-derived cultured cells that solve the difficulties of lipofection and provide a method for their production. This discovery will greatly contribute to the development of veterinary research.

## Results

### BE cells showed high transfection efficiency using lipofection

**Figure 1:**
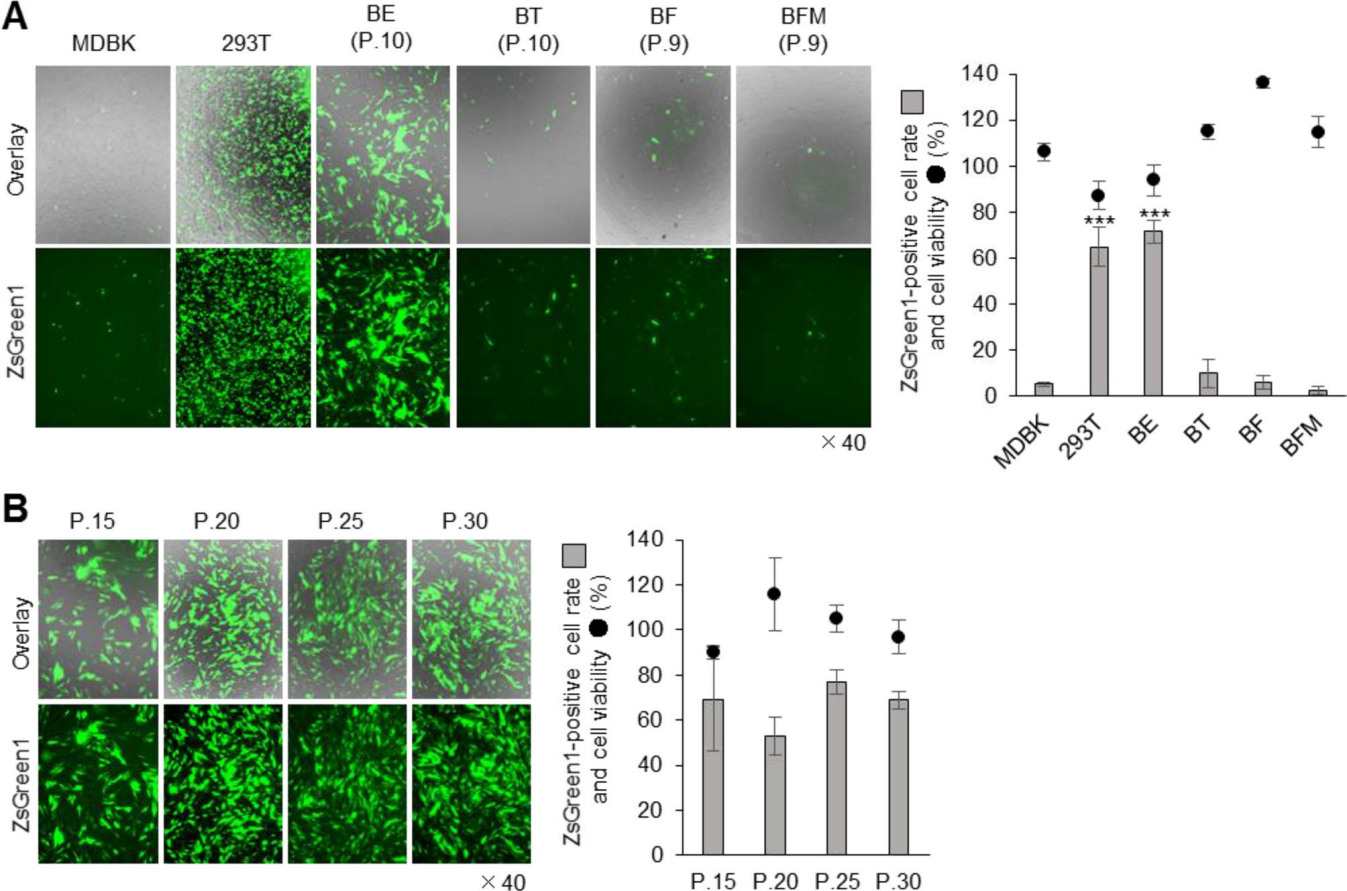
Transfection efficiency of lipofection in bovine-derived cultured cells. (**A**) MDBK, 293T, BE, BT, BF and BFM cells were seeded on a 24-well culture plate and transfected with 250 ng/well of ZsGreen1-expression vector, using the TransFectin Lipid Reagent. The transfected cells were observed under a fluorescence microscope at 48 h post transfection (hpt; left pictures). The ZsGreen1-positive cell rate and cell viability were measured at 48 hpt (right graph). Asterisks indicate significant differences from the control MDBK cells (***P < 0.001). (**B**) One BE cell clone could be passaged 30 times and maintained high lipofection efficiency. The transfection assay was described for (**A**).

To confirm the lipofection efficiency in bovine-derived cultured cells, we prepared primary cultured cells from several bovine tissues. Bovine primary cultures were derived from the BE, bovine testis (BT), ear tissue [as bovine fibroblasts (BF)] and bovine foetal muscle (BFM). Bovine kidney-derived MDBK cells were selected as a representative, established cell line. The TransFectin Lipid Reagent (Bio-Rad, Hercules, CA), which is considered suitable for bovine-derived cultured cells, was selected as the lipofection reagent^14^. Cultured 293 T cells, which are known to be suitable for efficient gene introduction^15^, showed high lipofection efficiency (64.8% ± 8.5%); however, the efficiency was low in BT (10.0% ± 6.1%), BF (6.1% ± 3.0%), BFM (2.3% ± 1.7%) and MDBK cells (5.3% ± 1.0%). In contrast, BE cells showed significantly higher lipofection efficiency (71.5% ± 4.9%; Fig. 1A). Surprisingly, BE cells could be passaged 30 times (passage number 30; P.30) and continued to maintain high lipofection efficiency from P.15 to P.30 (Fig. 1B).

### Characterisation of BE cells

**Figure 2:**
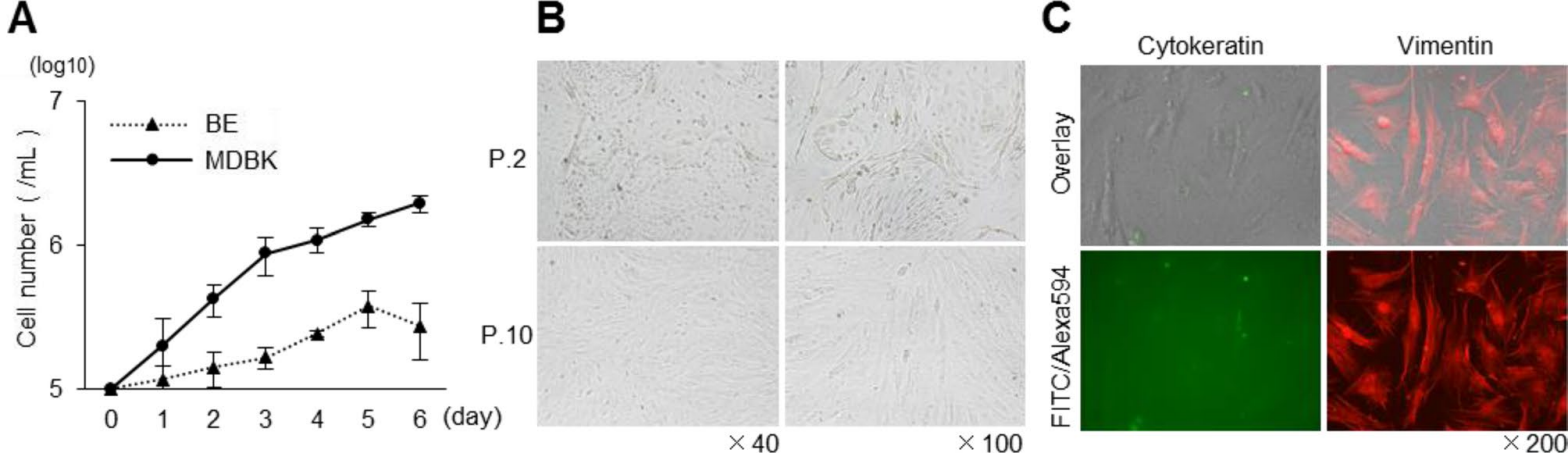
Characterisation of BE cells. (**A**) The growth kinetics of BE cells (P.20) were compared with those of MDBK cells. Both cell types were seeded at a concentration of 1 × 10^5^ cells/well, and the number of cells was counted every 24 h for 6 days. (**B**) BE cells pictured from P.2 and P.10. (**C**) IFA, using anti-cytokeratin and anti-vimentin antibodies, was performed to investigate the constituent cells in the BE cells (P.10).

To better understand the properties of BE cells suitable for lipofection, we characterised the cells. The proliferation of BE cells was slower than that of MDBK cells, and the doubling time on days 1–5 of culture was calculated to be 62.65 ± 22.42 h for BE cells and 34.47 ± 8.71 h for MDBK cells (Fig. 2A). In the BE cells from P.2, cells with several different shapes could be observed (Fig. 2B, upper pictures); however, after a series of three-fold expansion passages at 5-day intervals, the cells homogenised into a spindle-shaped population at P.10 (Fig. 2B, lower pictures). To investigate the constituent cells found in BE cell cultures from P.10, we performed indirect fluorescent antibody (IFA) method, using anti-cytokeratin and anti-vimentin antibodies. The BE cells of P.10 were found to be negative for cytokeratin- and positive for vimentin-expression (Fig. 2C). We designated this population of cells suitable for lipofection as BE-1 cells.

### Artificially immortalised iBE-1 cells also have good lipofection efficiency

**Figure 3:**
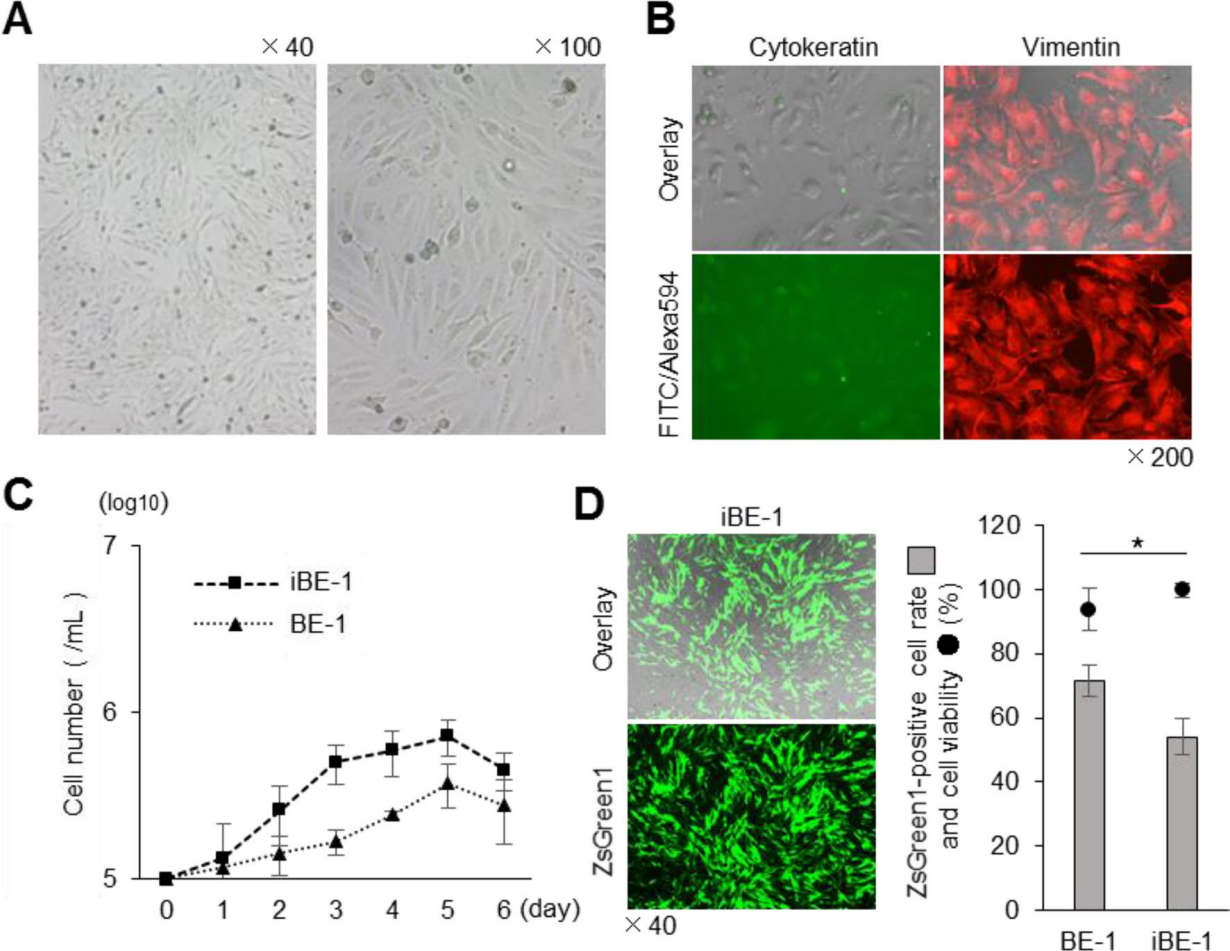
Characterisation of artificially immortalised iBE-1 cells. (**A**) Pictures of iBE-1 cells from P.9–1. (**B**) IFA was performed to investigate the constituent cells in iBE-1 cells (P.9–25). (**C**) The growth kinetics for iBE-1 cells (P.9–25) were compared with those for BE-1 cells (P.20). These cultured cells were seeded at a concentration of 1 × 10^5^ cells/well, and the number of cells was counted every 24 h for 6 days. (**D**) ZsGreen1-expression vector (250 ng/well) was transfected into iBE-1 cells (P.9–36), using the TransFectin Lipid Reagent, and the ZsGreen1-positive cell rate and cell viability were measured at 48 hpt. The lipofection efficiency and cell viability of BE-1 cells is shown in Fig. 1A. Asterisks indicate significant differences between BE-1 and iBE-1 cells (*P < 0.05).

To enhance the ease of using BE-1 cells, we attempted to produce an established cell line that was suitable for lipofection. We artificially immortalised the BE-1 cells by the introduction of the SV40 large T antigen, generating iBE-1 cells (Fig. 3A). We then characterised the iBE-1 cells. The IFA method confirmed that the expression of cytokeratin and vimentin in iBE-1 cells was similar to that in the parental cells (Fig. 3B). The iBE-1 cells grew faster than the parental BE-1 cells, with a doubling time of 38.94 ± 9.10 h for the iBE-1 cells (Fig. 3C). To confirm whether the lipofection efficiency was retained, we performed transfection assays using the TransFectin Lipid Reagent. The iBE-1 cells demonstrated a lipofection efficiency of 54.0% ± 5.5% (Fig. 3D). Although the iBE-1 cells were slightly less efficient than the parental BE-1 cells, they were able to grow more efficiently than the parental BE-1 cells while maintaining a lipofection efficiency of more than 50%.

### Conditions of BE cells suitable for lipofection

#### Differences in the oestrus cycle

**Figure 4:**
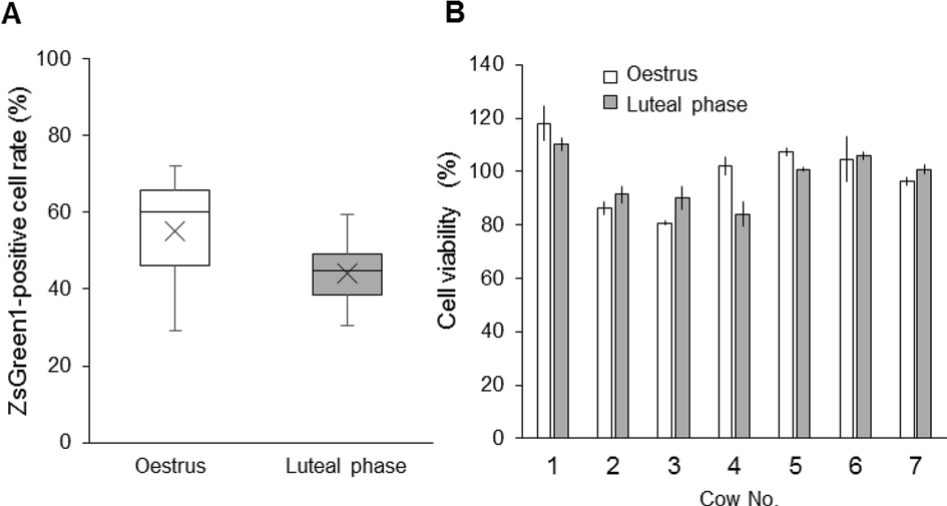
Comparison of lipofection efficiencies between BE-1 cells derived from oestrus and luteal phase. (**A**) BE-1 cells were prepared from seven cows in either the oestrus or the luteal phase. To compare the transfection efficiencies, ZsGreen1-expression vector (250 ng/well) was transfected into each set of BE-1 cells at P.5, using the TransFectin Lipid Reagent. The ZsGreen1-positive cell rate was measured at 48 hpt. Boxes indicate the first, the median and the third quartiles; whiskers indicate the minimum and maximum. ( ×) indicates the average value for all seven cows. There were no significant differences between the oestrus and luteal phase (*P* = 0.706). (**B**) Cell viabilities were measured at 48 hpt.

The BE cells, shown in Figs. 1 and 2, were derived from the BE at the luteal phase. To investigate whether differences in the oestrus cycle affected the lipofection efficiency of BE-1 cells, BE samples were collected at the oestrus and luteal phases, from seven cows, and cultured cells were produced from each sample. Cultured cells were used at P.5 for transfection assays using the TransFectin Lipid Reagent, and the lipofection efficiencies (Fig. 4A) and cell viabilities (Fig. 4B) were calculated. In the samples collected at oestrus, BE-1 cells from five cows showed lipofection efficiencies of 50% or higher; however, the cells derived from one cow showed an efficiency of 30% or lower. In samples collected at the luteal phase, only two cows showed lipofection efficiencies of 50% or higher, whereas the efficiencies of the other five cows ranged from 30.4 to 47.8%. The average efficiencies of samples derived from the oestrus and luteal phase samples were 54.98 ± 15.93% and 44.20 ± 10.02%, respectively, and there was no significant difference between them (*P* = 0.0706).

#### Differences in the constituent cell populations among BE cells

**Figure 5:**
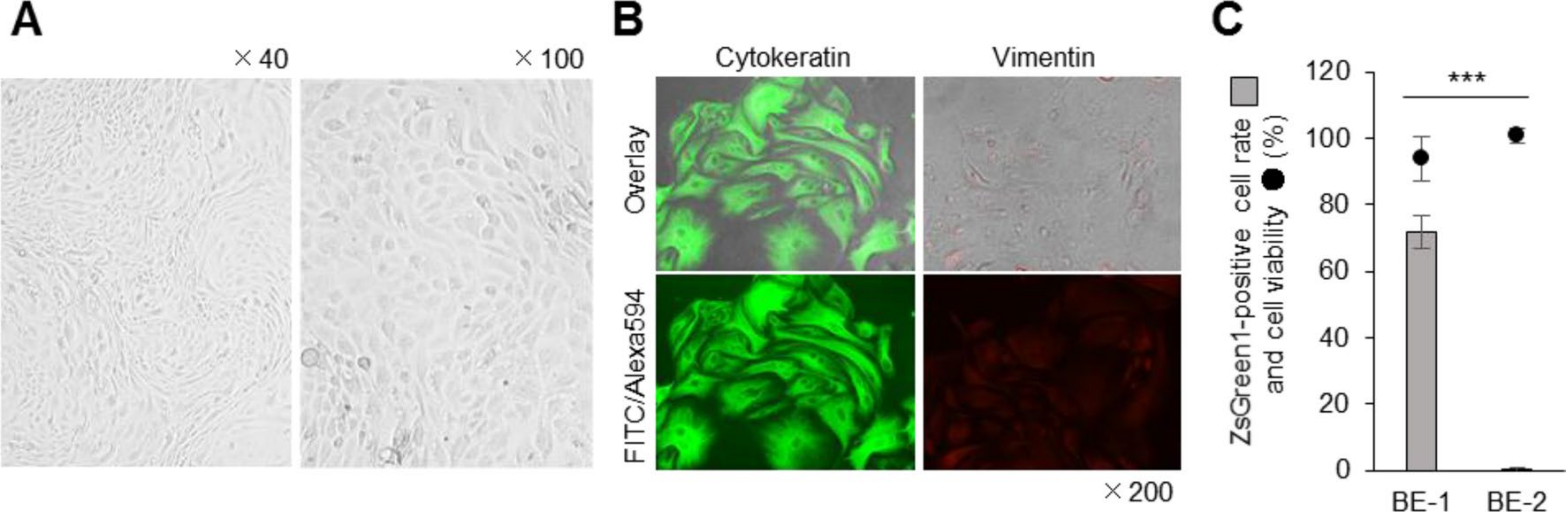
BE-2 cells were not suitable for lipofection. (**A**) BE-2 cells (P.10) were obtained by performing three-fold expansion passaging at 10‒14-day intervals. The cells were derived from the endometrium at oestrus. (**B**) The IFA method was used to check the constituent cells. (**C**) ZsGreen1-expression vector (250 ng/well) was transfected into BE-2 cells (P.10), using the TransFectin Lipid Reagent, and the ZsGreen1-positive cell rate and cell viability were measured at 48 hpt. The lipofection efficiency and cell viability of BE-1 cells is shown in Fig. 1A. Asterisks indicate significant differences between BE-1 and BE-2 cells (***P < 0.001).

In Fig. 2B, the BE-1 cells consisting of spindle-shaped cell population was obtained by three-fold expansion passages at 5-day intervals. On the other hand, three-fold expansion passages at 10–14-day intervals resulted in round-shaped cell population as shown in Fig. 5A. We designated this population as BE-2 cells and investigated whether they were also suitable for lipofection. The constituent cells were confirmed by the IFA method and the BE-2 cells were positive for cytokeratin- and negative for vimentin-expression (Fig. 5B). When transfection assays using the TransFectin Lipid Reagent were performed, the lipofection efficiency was significantly low in the BE-2 cells when compared to that of BE-1 cells (0.3 ± 0.6%; Fig. 5C).

These results revealed that (1) differences in the oestrus cycle do not significantly affect the lipofection efficiency of the BE-1 cells and (2) BE-2 cell populations positive for cytokeratin- and negative for vimentin-expression, obtained by passaging at long intervals, are not suitable for lipofection.

### Applications of BE-1 and iBE-1 cells

**Figure 6:**
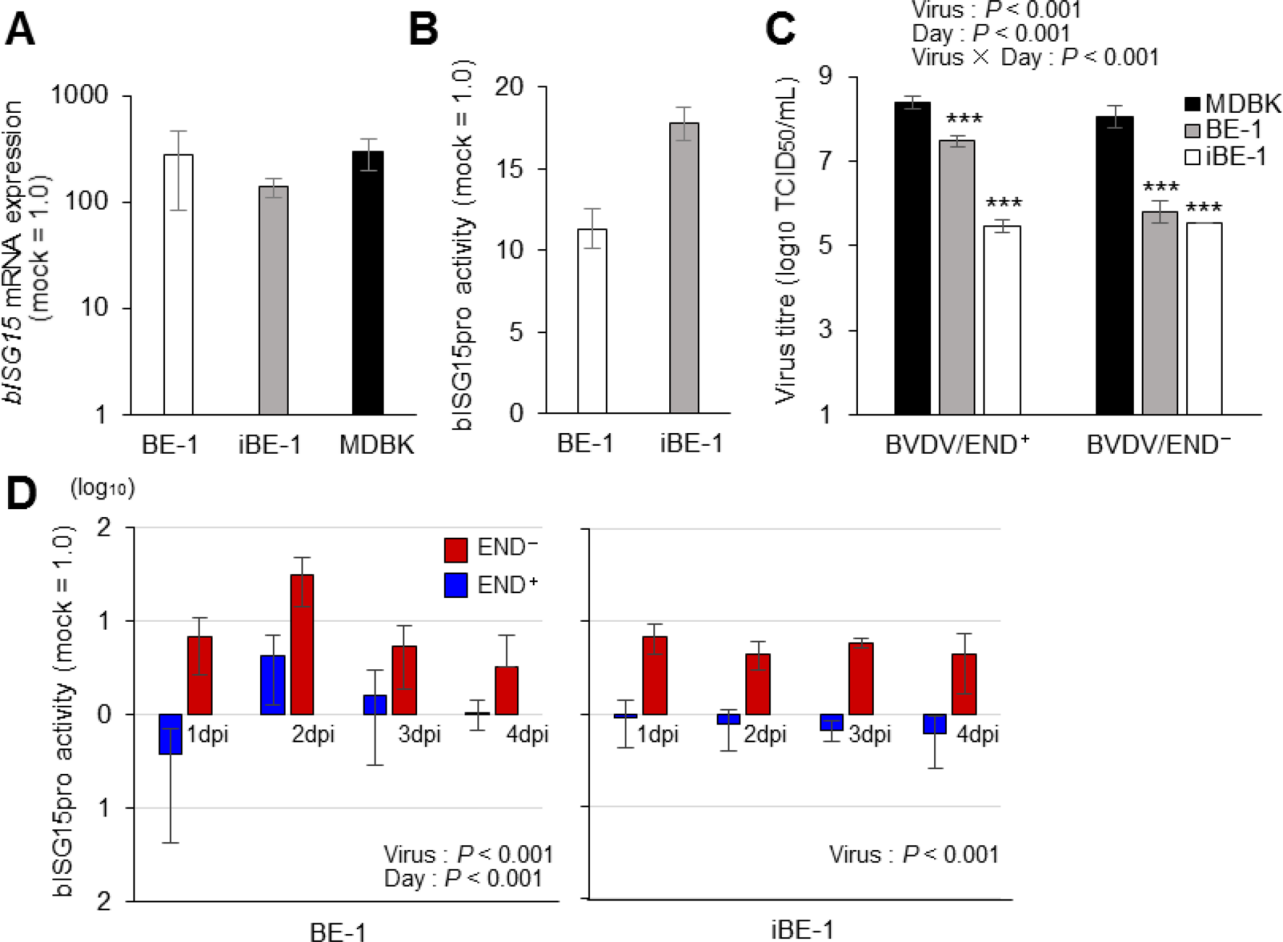
Applications of BE-1 and iBE-1 cells. (**A**) The expression of *bISG15* mRNA in BE-1, iBE-1 and MDBK cells was measured by qPCR, 24 h after hrIFN-α (10 ng) treatment. No significant differences were observed in comparison with MDBK cells. (**B**) Reporter plasmids were introduced into BE-1 and iBE-1 cells, using the TransFectin Lipid Reagent, and hrIFN-α (10 ng) was added to the transfected cells at 24 hpt. Luciferase activities were measured 24 h after hrIFN-α stimulation. (**C**) To investigate the susceptibility of BE-1 and iBE-1 cells to BVDVs, BVDV/END^+^ and BVDV/END^–^ were inoculated into these cells, at an MOI of 1.0. MDBK cells were used as the positive control. Viral titres were calculated using a peroxidase-linked assay, with a pestivirus-specific antibody. Statistical analysis was performed for each virus type and compared with the titres calculated in MDBK cells. (**D**) Reporter plasmids were introduced into BE-1 and iBE-1 cells, and BVDV/END^+^ or BVDV/END^–^ was inoculated into the cells at 24 hpt. Luciferase activities were measured 1‒4 days after viral inoculation, and bISG15 promoter activities were calculated.

To confirm whether BE-1 and iBE-1 cells could be applied to biological assays, a luciferase-based reporter assay was performed. We first constructed a reporter plasmid that can measure the promotor activity of bovine interferon-stimulated gene 15 (bISG15) and attempted to measure activity by introducing the reporter and control plasmids into each cells. Before measuring the bISG15 promoter activity, it was confirmed using quantitative polymerase chain reaction (qPCR) whether the induction of *bISG15* mRNA expression occurs in both cells. As a result, *bISG15* mRNA expression was increased in both cells at 24 h after human-recombinant interferon (hrIFN)-α stimulation (Fig. 6A). When hrIFN-α was added to reporter plasmids-transfected BE-1 and iBE-1 cells and the promoter activity was measured at 24 h later, the results showed that bISG15 promoter activity increased in both cell types (Fig. 6B). Promoter activity was observed in response to hrIFN-α in BE-1 (11.31 ± 1.2-fold) and iBE-1 (17.7 ± 1.0-fold) cells, as expected, indicating that the reporter plasmids were introduced effectively into both cell types. We next determined whether this reporter assay could be used to detect innate immune responses induced by viral infections. The expression of *bISG15* mRNA can be suppressed and induced by infection with the bovine viral diarrhoea viruses (BVDV)/END^+^ and BVDV/END^–^, respectively^16^. Therefore, these viruses were used to infect the reporter plasmid-transfected BE-1 and iBE-1 cells, and promoter activities were measured 1–4 days after infection. When the susceptibility to BVDVs was examined prior to performing the reporter assay, both cell types were found to be sensitive to BVDVs, although they were both less sensitive than MDBK cells (Fig. 6C). Increased bISG15 promoter activities were observed in both cell types during BVDV/END^–^ infection, and the promoter activities were suppressed in both cell types during BVDV/END^+^ infection, indicating that promoter activities were regulated by each BVDV infection, as expected (Fig. 6D). These results showed that BE-1 and iBE-1 cells can be used for reporter assays.

### BE-1 and iBE-1 cells can be transfected efficiently with various lipofection reagents

**Figure 7:**
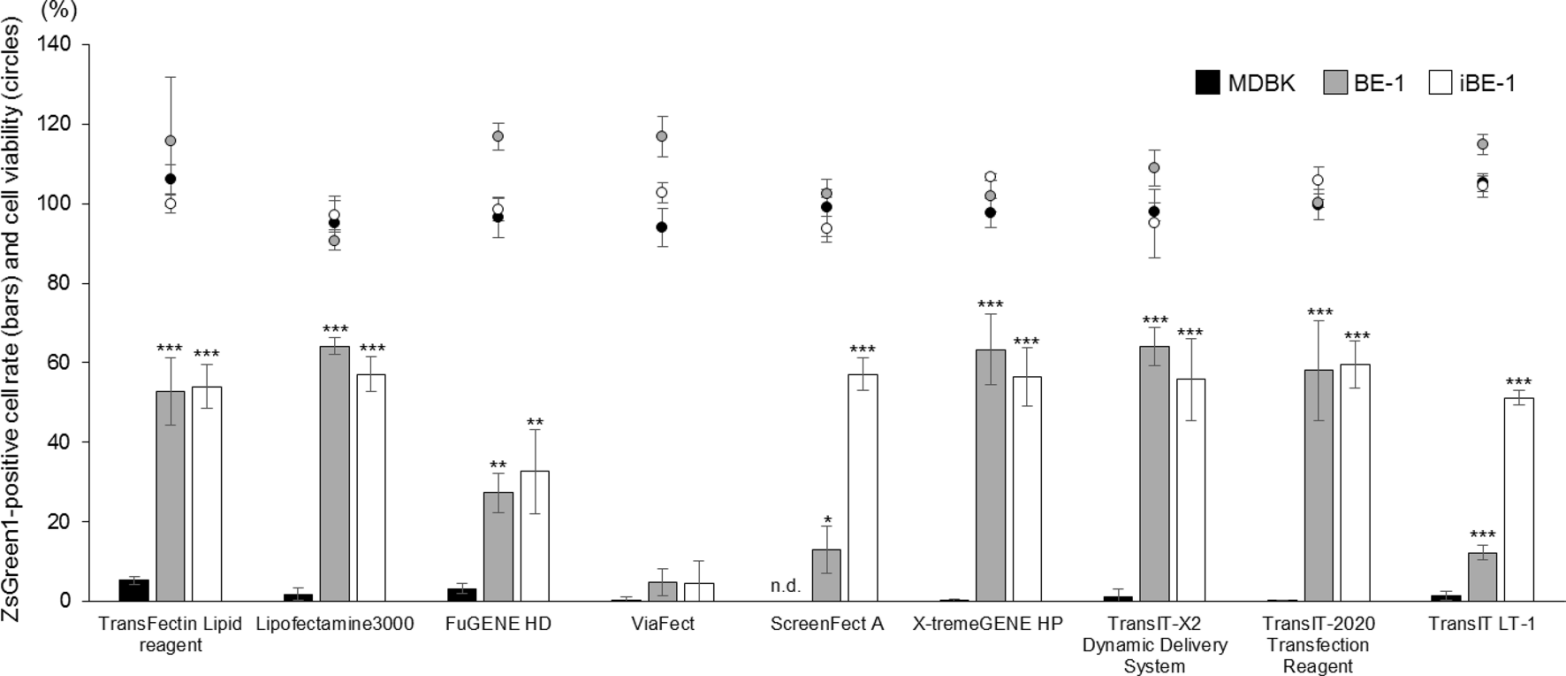
Comparison of transfection efficiencies using various lipofection reagents in BE-1 and iBE-1 cells. The ZsGreen1-expression vector (250 ng/well) was transfected into MDBK cells at P.135, BE-1 cells at P.20‒24 and iBE-1 cells at P.9–5‒7, using nine lipofection reagents. The ZsGreen1-positive cell rate (bars) and cell viability (circles) were measured at 48 hpt. ‘n.d.’ means ‘not detected’. Asterisks indicate significant differences from the efficiency of MDBK cells in each lipofection reagent (***P < 0.001, ** *P* < 0.01, *P < 0.05).

Finally, using BE-1 and iBE-1 cells, we confirmed whether reagents other than the TransFectin Lipid Reagent could be used for efficient lipofection (Fig. 7 and Supplementary Fig. S1). MDBK cells were used as a comparative control, and the BE-1 cells used were from P.20 to P.24, which demonstrate stable cell growth. The lipofection efficiencies for MDBK cells were low with all the reagents tested, and even the highest efficiency was only 5.3 ± 1.0%, which was observed with the TransFectin Lipid Reagent. In BE-1 cells, five reagents, excluding FuGENE HD, ViaFect, ScreenFect A and *Trans*IT-LT1, showed lipofection efficiencies of 50% or higher, and the highest efficiency was observed when Lipofectamine 3000 was used (64.2 ± 2.2%). The iBE-1 cells showed results similar to those for the BE-1 cells, and the lipofection efficiencies of ScreenFect A and *Trans*IT-LT1 were improved compared to that of the BE-1 cells. In the iBE-1 cells, the highest efficiency was observed when *Trans*IT-2020 was used (59.6 ± 6.0%).

## Discussion

This study has shown that cell population as BE-1 cells derived from BE cells show a high transfection efficiency using lipofection. This cell population was obtained by passaging at short intervals and was also found to show negative expression of cytokeratin and positive expression of vimentin. The high lipofection efficiency was maintained even after artificial immortalisation, and a more user-friendly version of iBE-1 cells could be produced.

In the present study, it was revealed that BE-1 cells showed a high transfection efficiency using lipofection. As shown in Fig. 2B, the BE cells of P.2 were mixed with various shapes of cells, and spindle-shaped (stromal-like) and round-shaped (epithelial-like) cells were present in various proportions^17^ (Fig. 2B). When the BE cells were passaged with three-fold expansion at intervals of 4‒5-days, the spindle-shaped cell population (BE-1) became dominant (Fig. 2B and C). In contrast, spindle-shaped cells disappeared and round-shaped cell population (BE-2) became dominant when the cells were passaged with three-fold expansion at intervals of 10‒14-days (Fig. 5A and B). BE-2 cell population was found to have extremely low lipofection efficiency (Fig. 5C). When preparing cultured cells from the BE, the culture must be maintained carefully because the constituent cell populations can change depending on the cell passage timings. In this study, BE-1 and BE-2 cells were cultured initially in a mixed state (Fig. 2B); however, methods to separate the cells have also been reported^18,19^. Kelly et al.^18^ reported a separation method using filtration and Murakami et al.^19^ reported a culturing method by excluding epithelial cells. We also attempted to separate BE-1 cell populations by filtration methods using BE cells in early passages. As a result, we were able to recover BE-1 cell population by culturing the cell suspension that had passed through the filter (40 µm-pore size) (data not shown). The method of separating cell populations according to the timing of passaging takestime, so the filtration method may be effective if you want to obtain BE-1 cells as soon as possible. To summarise, the methods used to prepare BE-1 cells were as follows: (1) endometrial samples were collected from live cows (as differences in the oestrus cycle do not significantly affect the lipofection efficiency of BE-1 cells, endometrial samples may be taken from either the oestrus or luteal phase.); (2) BE cells were cultured (see Materials and Methods); (3) BE cells were passaged with three-fold expansion at 4‒5-day intervals, allowing spindle-shaped cell population; BE-1 to become dominant. By performing steps (1) to (3), BE-1 cells can be obtained easily and applied to transfection assays using lipofection.

Generally, lipofection is viewed as being more difficult to perform in primary cultured cells than in established cell lines^20^. Surprisingly, BE-1 cells did not match this trend. Despite being primary culture cells, at least one BE-1 cell clone could be passaged more than 30 times, while maintaining high transfection efficiency (Fig. 1B). However, unfortunately, BE-1 cells similar to those shown in Fig. 1B are unlikely to be obtained. The spontaneous immortalisation of primary cultured cells is a very rare event^21^, and most BE-1 cells could be passaged fewer than ten times. If the maintenance of BE-1 cells for long periods is desired, cells should be collected from the luteal phase. When comparing BE-1 cells obtained at the oestrus and luteal phase, as shown in Fig. 4, most of the oestrus-derived BE-1 cells stopped growing at approximately P.5‒7, whereas four of seven luteal phase-derived BE-1 cells could be passaged more than ten times (data not shown). Additionally, the BE cells shown in Fig. 1B were produced from endometrial samples acquired at the luteal phase. Differences in the oestrus cycle do not significantly affect the lipofection efficiency of BE-1 cells (Fig. 4A), but may affect the cell lifespan, so the timing of samples obtained from the endometrium must be considered, according to the purposes of the experiment.

Since most reporter assays require the introduction of multiple DNAs into the cells, it is essential to use cells with high lipofection efficiency. However, as there were no bovine-derived cultured cells that could be used for efficient gene transfer by lipofection, we were forced to use cells of non-bovine animal origin (e.g. 293 T cells) only for reporter assays. Therefore, it is questionable whether the assays accurately reflected the reactions occurring in the bovine cells. In the present study, it was indicated that the BE-1 and iBE-1 cells could be used in a reporter assay (Fig. 6B and D). Therefore, it will be possible to analyse in more detail the innate immune response and metabolic activities of bovine-derived cells, which have previously been obscure. Facilitating gene transfer by lipofection will enable not only reporter assays, but also genetic modification of cultured cells. It is also expected to enable a more efficient use of genome editing technologies such as the CRISPR/Cas system. Furthermore, it will be possible to synthesise bovine-derived recombinant proteins, with potential in the development of biopharmaceuticals. In addition, BVDV is a typical factor that contaminates bovine serum and cultured cells^22,23^; however, the BE-1 and iBE-1 cells used in this study displayed no BVDV contamination (data not shown). Furthermore, both cells are susceptible to viruses that infect cattle, other than BVDV (Supplementary Fig. S2), and may be applicable to basic research and inspection of some viruses.

It was difficult to clarify from the current results why BE-1 cell populations are suitable for lipofection. The characteristics of BE-1 cells, such as the ability to allow expanded passages (up to fivefold) and long lifespan of the cells, may be responsible for the ease of lipofection^24^. The lipid metabolic activity of the endometrial tissue and composition of the lipofection reagent (lipid-based or non-lipid-based)^24^ may also play a role in the lipofection efficiency, but this study did not provide a unified view. Further characterisation of BE-1 and BE-2 cells and identification of cell types may help to clarify the factors that determine lipofection efficiency in cultured cells of bovine origin.

## Materials and methods

### Animals

All the experimental procedures complied with the Guidelines for the Care and Use of Animals established by Nippon Veterinary and Life Science University and all animal protocols were approved by the Institutional Animal Care and Use Committee (Nippon Veterinary and Life Science University [Tokyo, Japan], No. 2020S-44). All animal experiments were performed in accordance with ARRIVE guidelines (https://arriveguidelines.org).

In the present study, eight healthy lactating Holstein dairy cows were used (parity: 2.0 ± 1.5, mean ± SD). They were housed at the Fuji Animal Farm. Healthy cows were defined as follows: none of the cows used in this study were diagnosed with clinical ketosis, hypocalcaemia, metritis, retained placenta, or displaced abomasum after normal parturition, as assessed by a veterinarian. In addition, they did not suffer subclinical endometritis, which was detected by the cytological evaluation of the endometrium. To synchronise the oestrous cycle, oestrous synchronisation programme was performed. Briefly, oestradiol benzoate (2 mg, ASKA Animal Health Co. Ltd, Tokyo, Japan) was treated intramuscularly (i.m.), and a progesterone preparation (CIDR, Zoetis Japan, Tokyo, Japan) was inserted the into the vagina (Day -11). After 9 days, prostaglandin F_2_α (PGF_2α_, 25 mg i.m., Veterinary Pronalgon F, Zoetis Japan) was treated to induce luteolysis (Day -2) and gonadotropin-releasing hormone (GnRH, 100 µg i.m., Fertireline, Fujita Pharmaceuticals Co. Ltd, Tokyo, Japan) was treated to induce ovulation (= Day 0) on 2 days after PGF_2α_ administration. Endometrial samples were collected on Day 0 and 7 of the oestrous cycle. On average, Day 0 sampling was performed 143.0 ± 82.5 days (103–328 days) after parturition, and mean milk yield per cow was 31.6 ± 7.3 kg (25.6–46.5 kg).

### Collection of uterine endometrial tissue

Endometrial tissue was collected from uterine body using the endometrial biopsy technique. Endometrial tissue was obtained using endometrial forceps manufactured specifically for uterine tissue collection in large animals (Endometrial biopsy gun, Fujihira Industry Co., Ltd., Tokyo, Japan). Before uterine endometrial tissue collection, the cows’ vulva and perineal area were cleaned to avoid contamination. The biopsy gun was introduced through the vagina, advancing until the external cervical orifice. The biopsy gun was guided by transrectal palpation through the cervix and advanced into the uterine body. After ensuring the placement of the tip of the biopsy gun at the uterine body, the inner sheath of biopsy gun was pulled by retracting the rod. The uterine wall was pressed gently into the lateral opening region of outer sheath of the biopsy gun using the thumb by transrectal palpation. Endometrial tissue was clipped shut by closing the lateral opening region by the inner sheath pushing the rod back to the initial position. Tissues were taken from three different endometrial areas for reducing the bias of tissue characteristics. We did not distinguish the endometrial tissue by caruncle and intercarunclar area. The biopsy gun was then withdrawn, and the endometrial tissue was collected by tweezers, and placed in phosphate buffered saline (PBS) solution.

### Cells and viruses

Endometrial samples were placed in a 0.25% trypsin-PBS solution and warmed at 37 °C for 1 h. After the addition of culture medium [Eagle’s Minimum Essential Medium (EMEM), containing 10% BVDV-free foetal bovine serum (FBS) and antibiotics (100 IU/mL penicillin, 100 µg/mL streptomycin, 2 µg/mL gentamicin and amphotericin B)], samples were pipetted well and centrifugation was performed. After removing the supernatant, fresh medium was added to the cell, and the cells were seeded in culture flasks. The cells in flasks were defined as BE cells at P.0.

A monolayer of BE-1 cells (P.9) in six-well plates were transfected with plasmid encoding the SV40 large T antigen (2 µg/well), using the TransFectin Lipid Reagent. Two days after incubation, the medium was replaced with medium containing 200 µg/mL G418. The cells were then cultured for 2‒3 weeks, and all surviving cells were transferred to a 25-cm^2^ flask. The cells in the flask were defined as iBE-1 cells (P.9–0).

BF cells were produced from the ear tissue of Holstein and were prepared according to the method described by Oliveira et al.^13^ BT and BFM cells were also prepared from Holstein. MDBK cells were cultured in EMEM, containing 0.295% tryptose phosphate broth, 1% FBS, 1% horse serum, and antibiotics (100 IU/mL penicillin and 100 µg/mL streptomycin). Human foetal kidney-derived 293 T cells purchased from American Type Culture Collection (ATCC, CRL-3216) were cultured in Dulbecco’s Modified Eagle Medium, containing 3% FBS, 4% horse serum and antibiotics (100 IU/mL penicillin and 100 µg/mL streptomycin). All cultured cells were incubated under conditions of 37 °C and 5% CO_2_ supply.

The non-cytopathogenic BVDV/END^+^ and /END^−^ were generated by reverse genetics^16^.

### Antibodies, reagents and plasmids

Lipofection were performed using the *Trans*IT-LT1 Transfection Reagent, *Trans*IT-X2 Dynamic Delivery System and *Trans*IT-2020 Transfection Reagent (Mirus Bio, Madison, WI), TransFectin Lipid Reagent (Bio-Rad), Lipofectamine 3000 (Thermo Fisher Scientific, Waltham, MA), FuGENE HD and ViaFect (Promega, Madison, WI), ScreenFect A (FUJIFILM Wako, Osaka, Japan) and X-tremeGENE HP DNA Transfection Reagent (Roche, Basel, Switzerland). 4′,6-Diamidino-2-phenylindole (DAPI) and hrIFN-α were purchased from Dojindo Laboratories (Kumamoto, Japan) and PBL Assay Science (Piscataway, NJ), respectively. An anti-vimentin rabbit polyclonal and an anti-cytokeratin pan mouse monoclonal antibody (AE-1/AE-3) were purchased from Proteintech (Chicago, IL) and Novus Biologicals (Centennial, CO), respectively. Alexa Fluor 594-conjugated goat anti-rabbit antibody and fluorescein isothiocyanate-conjugated anti-mouse IgG were used as secondary antibodies for the IFA method.

The bISG15-promoter sequence was amplified by PCR, using a genome extracted from the endometrium of Holstein^25^. The amplified sequence was inserted into the pNL2.1-[Nanoluciferase; Nluc/Hygro] vector (Promega). The control plasmid, encoding firefly luciferase (Fluc, pGL4.53), was purchased from Promega. ZsGreen1 and SV40 large T antigen (NC_001669.1) sequences were inserted into the pcDNA3.1-myc/His B vector (TaKaRa Bio, Shiga, Japan). The sequences were confirmed by Eurofins Genomics (Tokyo, Japan) and analysed using GENETYX software (https://www.genetyx.co.jp/version9.0.7; Genetyx, Tokyo, Japan).

### Lipofection and cell viability assay

Cultured cells (1 × 10^5^ cells/well) were seeded on 24-well plates and incubated for 24 h. Plasmid DNA and the lipofection reagent were mixed at a ratio of 1:3 (250 ng DNA : 0.75 µL of reagent/well), regardless of which reagent was used. The mixture was incubated for the appropriate time (TransFectin Lipid Reagent, *Trans*IT-LT1, *Trans*IT-X2, *Trans*IT-2020 and X-tremeGENE HP for 30 min; ScreenFect A for 20 min; Lipofectamine 3000 and FuGENE HD for 15 min; and ViaFect for 5 min), according to the manufacturers’ instructions, and the mixture was added to the cells. Some reagents induce cytotoxicity, so the transfected cells were washed three times with PBS and the medium was replaced with fresh medium 4‒6 h after transfection, regardless of the reagent or cell type. DAPI staining was performed 48 h after transfection, and the cells were observed under a fluorescent microscope. ZsGreen1-positive cells and total cells, assessed by DAPI staining, were counted in three randomly selected fields of view, and the ZsGreen1-positive cell rate [(number of ZsGreen1-positive cells/number of total cells) × 100] was calculated.

Forty-eight hours after transfection, a volume of CellTiter-Glo2.0 Reagent (Promega) equal to the supernatant volume was added to the cells. The total cell lysate was collected and the luminescence intensity was measured. The relative cell viability of the transfected cells was calculated by setting the viability of non-transfected cells to 100%.

### qPCR

Cultured cells (3 × 10^4^ cells/well) were seeded on a 96-well plate and incubated for 24 h. The cells were treated with 10 ng hrIFN-α and incubated for 24 h. For the quantification of *bISG15* mRNA, total RNA was extracted using the RealTime Ready Cell Lysis Kit (Roche). First-strand cDNA synthesis and qPCR were performed using the Transcriptor Universal cDNA Master Kit (Roche) and PowerUP SYBR Green PCR Master Mix (Thermo Fisher Scientific), respectively. The mRNA expression levels were normalised to the amount of bovine *GAPDH* in each sample and quantified using gene-specific primers, as described previously^26,27^. The expression levels were quantified using the ΔΔCt method.

### Viral culture and titration

MDBK, BE-1 and iBE-1 cells were inoculated with BVDV, at a multiplicity of infection (MOI) of 1.0, and each culture supernatant was harvested 4 days post infection (dpi). Titration was performed using the same cells used for viral production. A peroxidase-linked assay was performed to detect BVDV antigen-positive cells^28^. The tissue culture infectious dose was calculated following the method described by Kärber^29^.

### Reporter assay

BE-1 and iBE-1 cells (1 × 10^4^ cells/well) were seeded on a 96-well plate and incubated for 24 h. Reporter plasmids (1 ng of Nluc-plasmid and 50 pg of pGL4.53/well) were transfected using the TransFectin Lipid Reagent and incubated for 24 h. The cells were stimulated with hrIFN-α (10 ng) and BVDVs (MOI = 1.0). The luciferase activities were measured at appropriate time points using a Nano-Glo Dual-Luciferase Reporter Assay System (Promega). Nluc activity was standardised against that of Fluc.

### Statistical analyses

All statistical analyses were performed using EZR (version 1.50, Saitama Medical Center, Jichi Medical University, Saitama, Japan), which is a graphical user interface for R (The R foundation for Statistical Computing, Vienna, Austria). More precisely, it is a modified version of R commander designed to add statistical functions that are frequently used in biostatistics^30^.

ZsGreen1-positive cell rates among cells (BE, 293T, BT BF, BFM and MDBK), the amount of *bISG15* mRNA expressions among cells (BE-1, iBE-1 and MDBK), and ZsGreen1-positive cell rates among cells (BE-1, iBE-1 and MDBK) in each lipofection reagents were analysed using one-way ANOVA for determining the main effects of cell. When a treatment effect was significant, Dunnett's test was used as a multiple comparison test to detect significant difference between MDBK and other cells. Virus titre and bISG15 promoter activity were analysed using two-way ANOVA for determining the main effects of virus (END^+^and END^-^) and dpi or cell (BE-1, iBE-1, and MDBK) and their interaction. When a significant interaction between virus and cell was detected, Holm’s test was used as a multiple comparison test to detect significant differences between MDBK and other cells within viruses.

The results are expressed as the means ± SD. Significant differences between group means were determined using paired Student’s *t*-tests. *P*-value < 0.001 are indicated by ***, *P*-value < 0.01 are indicated by ** and *P*-value < 0.05 are indicated by *, those of which are statistically significant.

## Supplementary Information

Supplementary Information.
