# Supplementary material for: Bovine endometrium-derived cultured cells are suitable for lipofection
Source: Sci Rep. 2021 Aug 10;11:16207. doi: 10.1038/s41598-021-95848-0 (PMC8355132; doi:10.1038/s41598-021-95848-0)
Supplement: Supplementary file 1 — Supplementary Information. [file 41598_2021_95848_MOESM1_ESM.docx]

**Supplementary information**

**Bovine endometrium-derived cultured cells are suitable for lipofection**

Mai Shiokawa^1^, Ryotaro Miura^2^, Aki Okubo^1^, Yujiro Hagita^3^, Itaru Yoshimura^3^, Hiroshi Aoki^1*^

^1^ School of Veterinary Nursing and Technology, Faculty of Veterinary Science, Nippon Veterinary and Life Science University, Tokyo 180-8602, Japan

^2^ School of Veterinary, Faculty of Veterinary Science, Nippon Veterinary and Life Science University, Tokyo 180-8602, Japan

^3^ Fuji Animal Farm, Nippon Veterinary and Life Science University, 799 Fujigane, Fuji-Kawaguchiko-machi, Yamanashi 401-0338, Japan

*Corresponding author:

Hiroshi AOKI, DVM, Ph.D.

Laboratory of Microbiology and Infectious Diseases,

School of Veterinary Nursing and Technology, Faculty of Veterinary Science,

Nippon Veterinary and Life Science University,

1-7-1, Kyonancho, Musashino, Tokyo, 180-8602, Japan

E-mail: aokihir@nvlu.ac.jp

Tel: 81-422-31-4151, Fax: 81-422-33-2094


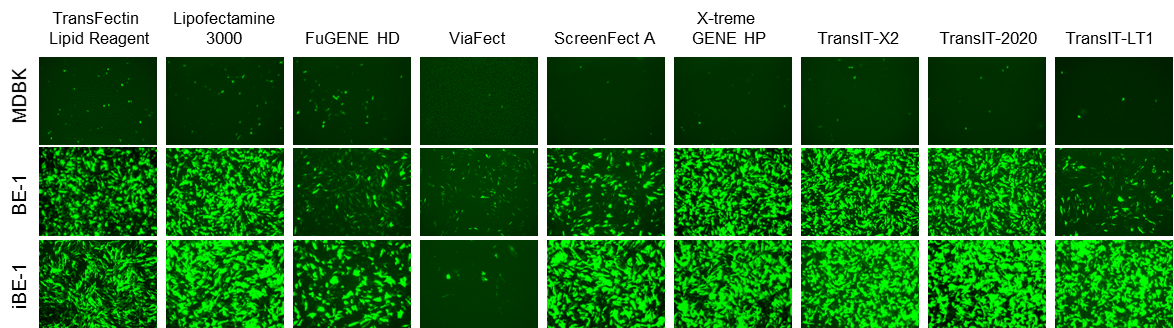
Supplementary figure. S1.

**Supplementary figure. S1. Comparison of transfection efficiency using various lipofection reagents in MDBK, BE-1 and iBE-1 cells.**

Transfection assay was performed in MDBK (P.135), BE-1 (P.20‒24) and iBE-1 (P.9-5‒7) cells using various lipofection reagents. The assay protocol is described in the Materials and Methods. The pictures (ZsGreen1, ×40) were taken 48 hours after transfection.


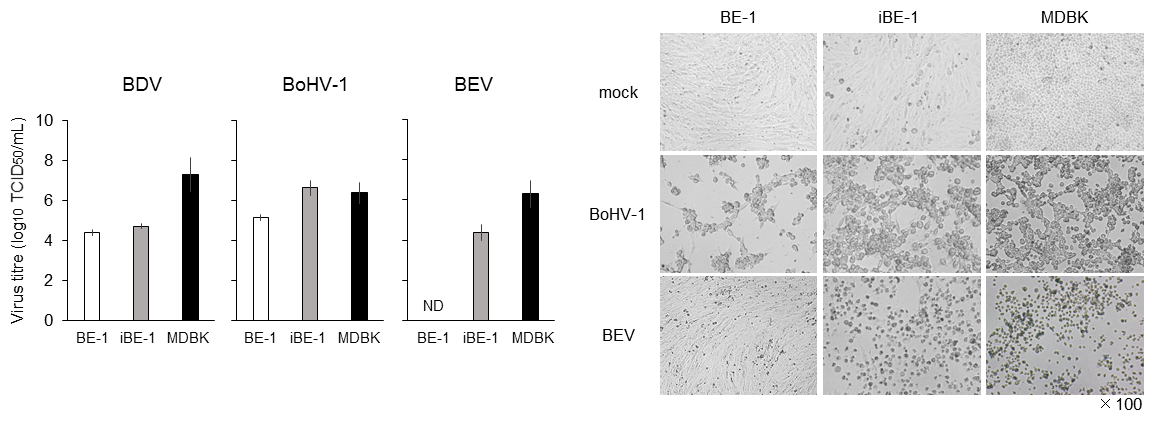
Supplementary figure. S2.

**Supplementary figure. S2. BE-1 and iBE-1 cells have susceptibility to viruses that can infect cattle.**

The susceptibility of both cells to the virus that infect with cattle was examined. The FNK2012-1 strain of border disease virus (BDV), the #758-43 strain of bovine herpesvirus-1 (BoHV-1), and the 2122 strain of bovine enterovirus (BEV) were inoculated into BE-1, iBE-1 and MDBK cells at an MOI of 1.0. Viral titration was performed using the same cells used for viral production (left graphs). BoHV-1 and BEV were cytopathogenic, and a cytopathic effect (CPE) was observed in each cell. Only CPE of BEV against BE-1 cells was weak (right pictures).
